# MiR-132-3p Regulates the Osteogenic Differentiation of Thoracic Ligamentum Flavum Cells by Inhibiting Multiple Osteogenesis-Related Genes

**DOI:** 10.3390/ijms17081370

**Published:** 2016-08-20

**Authors:** Xiaochen Qu, Zhongqiang Chen, Dongwei Fan, Chuiguo Sun, Yan Zeng

**Affiliations:** Department of Orthopaedics, Peking University Third Hospital, 49 North Garden Road, Haidian District, Beijing 100191, China; xiaochen13@hotmail.com (X.Q.); fdw_puth@163.com (D.F.); scg_puth@163.com (C.S.); zy_puth@163.com (Y.Z.)

**Keywords:** ossification of the ligamentum flavum, miR-132-3p, *FOXO1*, *GDF5*, *SOX6*, osteogenic differentiation

## Abstract

Ossification of the ligamentum flavum (OLF) is a disorder of heterotopic ossification of spinal ligaments and is the main cause of thoracic spinal canal stenosis. Previous studies suggested that miR-132-3p negatively regulates osteoblast differentiation. However, whether miR-132-3p is involved in the process of OLF has not been investigated. In this study, we investigated the effect of miR-132-3p and its target genes forkhead box O1 (*FOXO1*), growth differentiation factor 5 (*GDF5*) and SRY-box 6 (*SOX6*) on the osteogenic differentiation of ligamentum flavum (LF) cells. We demonstrated that miR-132-3p was down-regulated during the osteogenic differentiation of LF cells and negatively regulated the osteoblast differentiation. Further, miR-132-3p targeted *FOXO1*, *GDF5* and *SOX6* and down-regulated the protein expression of these genes. Meanwhile, *FOXO1*, *GDF5* and *SOX6* were up-regulated after osteogenic differentiation and the down-regulation of endogenous *FOXO1*, *GDF5* or *SOX6* suppressed the osteogenic differentiation of LF cells. In addition, we also found *FOXO1*, *GDF5* and *SOX6* expression in the ossification front of OLF samples. Overall, these results suggest that miR-132-3p inhibits the osteogenic differentiation of LF cells by targeting *FOXO1*, *GDF5* and *SOX6*.

## 1. Introduction

Ossification of the ligamentum flavum (OLF) is a rare disorder of heterotopic ossification of spinal ligaments that is almost exclusively reported in Eastern Asian countries. OLF primarily occurs in the thoracolumbar spine and is the main cause of thoracic spinal canal stenosis and myelopathy [1,2]. In the past several years, many studies have examined OLF development and progression at both histopathological and cellular levels. While these studies identified potential contributing factors, such as mechanical [3,4,5,6], metabolic [7,8], degenerative [9] and genetic factors [10,11], OLF development and progression continues to be inadequately understood.

One potential way that osteogenic differentiation is modulated is through microRNAs (miRNAs). MiRNAs, which comprise a substantial family of small (18–24 nucleotides), single-stranded non-coding RNAs, function in the regulation of mammalian cell gene expression. miRNAs regulate a target mRNA by binding its 3′-untranslated region (UTR) and subsequently mediating its degradation via the RNA-induced silencing complex (RISC) [12]. MiRNAs regulate a variety of physiological and pathological processes, with previous studies showing that particular miRNAs have the potential to positively or negatively regulate cells’ osteogenic differentiation and bone development [13,14]. For instance, miR-29b [15], miR-21 [16], miR-548d-5p [17], and miR-22 [18] were found to promote osteogenic differentiation and miR-92a [19], miR-214 [20], miR-30 [21], and miR-103a [22] were discovered to suppress osteogenesis, while miR-34a may have dimorphic effects [12,23]. Further, as reviewed by Huang C, studies on the miRNA revealed that miRNAs regulate osteogenesis by targeting osteoblast-related genes, particularly runt related transcription factor 2 (RUNX2) and Osterix, and targeting signaling pathways such as Wnt/β-catenin, transforming growth factor beta (TGF-β)/bone morphogenetic protein (BMP) and Notch [24].

The miR-212/132 family is highly conserved in vertebrates [25]. miR-132 and miR-212 are found in an intergenic region, exhibit similar mature sequences and share the same seed region, but the expression levels and the physiological functions of miR-132 and miR-212 are largely different [26]. Previous studies suggested that miR-132-3p can inhibit osteoblast differentiation and participate in the regulation of bone loss [27,28]. In addition, miR-132-3p was also found to regulate osteosarcoma [29,30,31,32], an osteogenic tumor that has the capability of osteoblast differentiation. However, whether miR-132-3p is involved in the process of OLF has not been investigated.

To explore the important role of miR-132-3p in thoracic ossification of the ligamentum flavum (TOLF), the present study investigated the effect of miR-132-3p and its target genes on the osteogenic differentiation of ligamentum flavum (LF) cells. Our results implied that miR-132-3p, which is down-regulated during osteogenic differentiation of LF cells, inhibits the differentiation process by targeting forkhead box O1 (*FOXO1*), growth differentiation factor 5 (*GDF5*) and SRY-box 6 (*SOX6*).

## 2. Results

### 2.1. MiR-132-3p Inhibits the Osteogenic Differentiation of Ligamentum Flavum (LF) Cells

**Figure 1:**
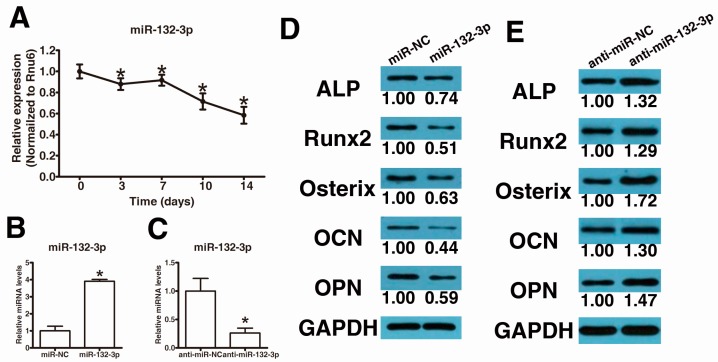

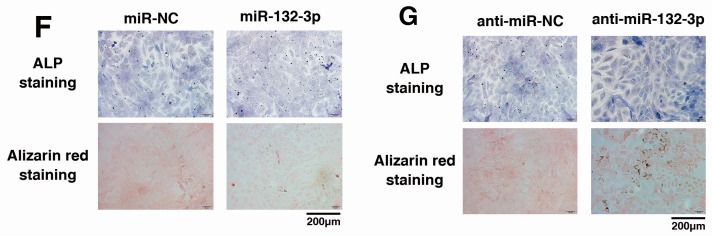
MiR-132-3p inhibits the osteogenic differentiation of ligamentum flavum cells. (**A**) Endogenous miR-132-3p expression levels were measured via quantitative real-time polymerase chain reaction (qRT-PCR) at different time points during osteogenic differentiation of ligamentum flavum cells; * *p* < 0.05 compared with day 0; (**B**) miR-132-3p expression was assessed via qRT-PCR in ligamentum flavum cells transfected with miRNA mimics; * *p* < 0.05 compared with nonspecific microRNA (miR-NC) group; (**C**) miR-132-3p expression was assessed via qRT-PCR in ligamentum flavum cells transfected with miRNA inhibitors; * *p* < 0.05 compared with anti-miR-NC group; (**D**,**E**) Western blot analysis of osteogenic marker protein expression after miR-132-3p overexpression (**D**) and reduction (**E**) at day 14; (**F**,**G**) alkaline phosphatase (ALP) staining and Alizarin Red staining at day 14 showed ALP activity and calcification after miR-132-3p overexpression (**F**) and reduction (**G**). Scale bar represents 200 µm and is fitted for every figure.

We first determined the temporospatial expression pattern of miR-132-3p in human LF cells cultured in osteogenic medium by qPCR to investigate the potential roles of miR-132-3p in the osteogenic differentiation of LF cells. Expression of miR-132-3p decreased at day 3 compared with that at day 0 and continuously decreased to day 14 (Figure 1A). This result suggests that miR-132-3p might negatively regulate the osteogenic differentiation of LF cells.

To further elucidate the role of miR-132-3p in the regulation of osteogenic differentiation of LF cells, synthetic mimics of miR-132-3p and inhibitors were transfected into LF cells, and the osteogenic capacity was examined. Intracellular miR-132-3p levels were markedly up-regulated by miR-132-3p mimics (Figure 1B) and substantially down-regulated by miR-132-3p inhibitors (Figure 1C). Furthermore, osteogenic differentiation was significantly inhibited after over-expression of miR-132-3p (Figure 1D,E) and significantly promoted after reduction of miR-132-3p (Figure 1F,G), as indicated by the expression changes of the osteogenic transcription factors, RUNX2 and Osterix, and osteoblastic markers, alkaline phosphatase (ALP), osteopontin (OPN) and osteocalcin (OCN), as well as ALP and Alizarin Red staining.

### 2.2. MiR-132-3p Directly Targets FOXO1, GDF5 and SOX6

**Figure 2:**
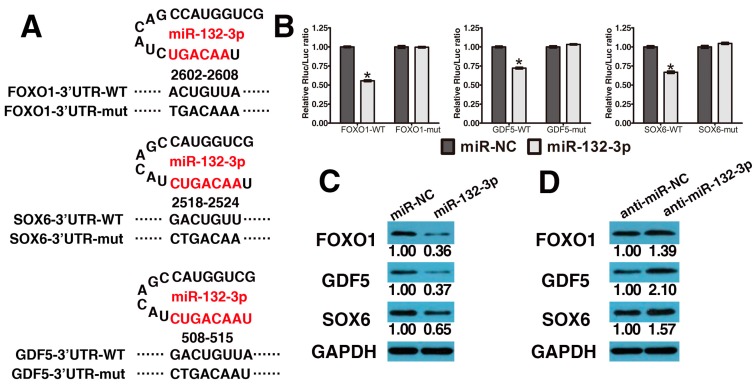
MiR-132-3p directly targets *FOXO1*, *GDF5* and *SOX6*. (**A**) The wild-type and mutated type miR-132-3p binding sites in the *FOXO1*, *GDF5* and *SOX6* 3′-UTR. The sequences in red font showed the blinding sites; (**B**) The wild-type (WT) 3′-UTR or mutant (MUT) 3′-UTR reporter plasmids of the three genes were co-transfected into HEK293T cells with either miR-132-3p or miR-NC and fluorescence was quantified; * *p* < 0.05 compared with miR-NC group; (**C**,**D**) *FOXO1*, *GDF5* and *SOX6* protein expression levels were examined via Western blot following miR-132-3p mimics (**C**) and inhibitors (anti-miR-132-3p) (**D**) transfection in ligamentum flavum cells.

To reveal the molecular mechanism by which miR-132-3p regulates the osteogenic differentiation of LF cells, TargetScan (http://www.targetscan.org/vert_71/) was utilized to forecast potential miR-132-3p targets. Among the candidates, we found that three osteogenesis-related genes, *FOXO1*, *GDF5* and *SOX6*, contain miR-132-3p binding sites in their 3′-UTRs. Next, we constructed luciferase reporters for each gene that contained either a wild-type (WT) 3′-UTR or a mutant (mut) 3′-UTR with mutant sequences of the miR-132-3p binding site (Figure 2A). The results showed that miR-132-3p repressed the luciferase activity of the 3′-UTR of each gene when compared to the nonspecific microRNA (miR-NC) control group, respectively. Additionally, no statistically significant alteration in luciferase activity was observed in the presence of the mutated 3′-UTR site (Figure 2B).

Next, we detected the gene expression of *FOXO1*, *GDF5* and *SOX6* after transfecting LF cells with the mimics of miR-132-3p and the inhibitor. We confirm that overexpression of miR-132-3p resulted in down-regulation of *FOXO1*, *GDF5* and *SOX6* in LF cells based on Western blot analysis (Figure 2C) and the reduction of miR-132-3p resulted in the opposite effects (Figure 2D). These results suggested that the effect of miR-132-3p during the osteogenic differentiation of LF cells is mediated by targeting these osteogenesis-related genes.

### 2.3. FOXO1, GDF5 and SOX6 Knockdown Inhibits Osteogenic Differentiation in LF Cells

**Figure 3:**
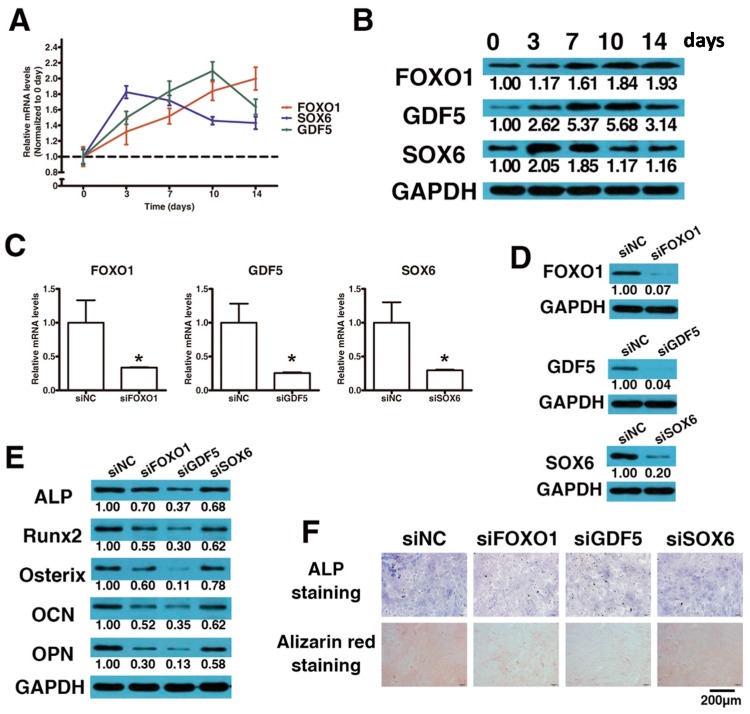
*FOXO1*, *GDF5* and *SOX6* knockdown inhibits osteogenic differentiation of ligamentum flavum cells. (**A**,**B**) *FOXO1*, *GDF5* and *SOX6* mRNA and protein expression levels examined via qRT-PCR and Western blot at different time points during osteogenic differentiation of ligamentum flavum cells; (**C**,**D**) *FOXO1*, *GDF5* and *SOX6* mRNA and protein expression level examined via qRT-PCR and Western blot following siRNAs transfection in ligamentum flavum cells; * *p* < 0.05 compared with non-targeting siRNA (si-NC) group; (**E**) Osteogenic marker protein expression examined via Western blot at day 14 after *FOXO1*, *GDF5* or *SOX6* knockdown; (**F**) ALP staining and Alizarin Red staining at day 14 showed inhibited ALP activity and calcification following *FOXO1*, *GDF5* or *SOX6* knockdown when compared with siNC group. Scale bar represents 200 µm and is fitted for every figure.

The mRNA and protein expression levels of *FOXO1*, *GDF5* and *SOX6* were determined by quantitative real-time polymerase chain reaction (qRT-PCR) (Figure 3A) and Western blot analyses (Figure 3B). All three were up-regulated in expression after osteogenic differentiation compared with day 0. Specifically, *FOXO1* was continually increased to day 14, while *GDF5* and *SOX6* were decreased (still higher than day 0) after day 10 and 7, respectively.

To examine the functional effects of *FOXO1*, *GDF5* and *SOX6* on the osteogenic differentiation of LF cells, siRNA-induced mRNA knockdown for each gene was employed and it significantly reduced both mRNA and protein expression of *FOXO1*, *GDF5* and *SOX6* (Figure 3C,D). Furthermore, *FOXO1*, *GDF5* or *SOX6* knockdown inhibited the osteogenic differentiation of LF cells, as indicated by reduced RUNX2, Osterix, ALP, OPN and OCN expression (Figure 3E) and reduced ALP and Alizarin Red staining (Figure 3F).

### 2.4. FOXO1, GDF5 and SOX6 Protein Expression in OLF Samples

**Figure 4:**
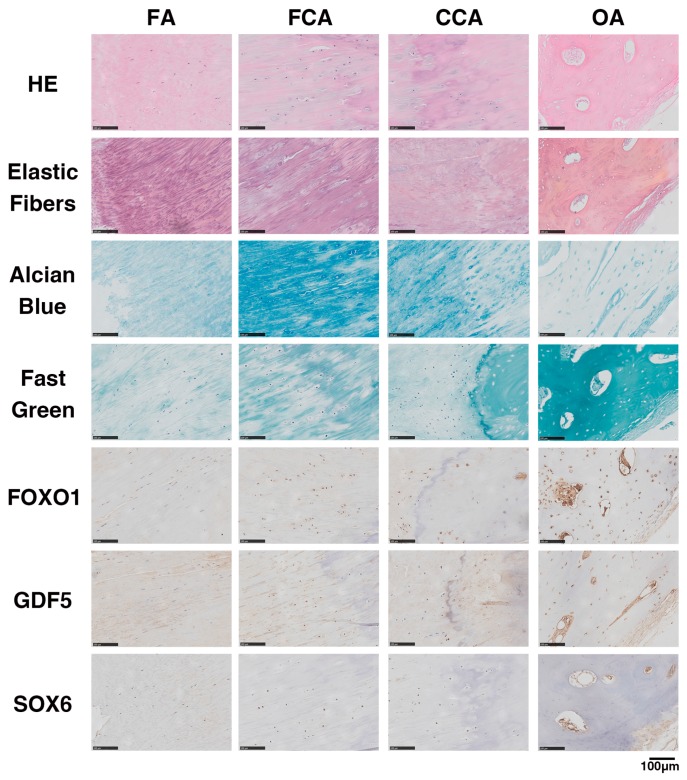
Representative hematoxylin-eosin staining, tissue-specific staining and immunohistochemical staining for *FOXO1*, *GDF5* or *SOX6* in OLF samples. Scale bar represents 100 µm and is fitted for every figure.

Structurally, pathological specimens of endochondral ossification exhibit an ossification front, including a fibrocartilage area (FCA) and calcified cartilage area (CCA), between the ossified area (OA) and ligamentous fiber area (FA). The HE staining, tissue-specific staining and immunohistochemical (IHC) staining in each area are shown in Figure 4. In FA, the Elastic Fibers staining showed positive and regular expression, and the IHC for *FOXO1*, *GDF5* and *SOX6* was negative. In FCA and CCA, the expressions of Elastic Fibers staining were decreased and irregular, and the Alcian Blue staining was positive. The IHC results suggested that *FOXO1*, *GDF5* and *SOX6* were positive in the round cells of the FCA and CCA. Further, in OA, the Fast Green staining was positive. IHC results showed that *FOXO1* and *GDF5* were strongly stained in the nuclei of osteoblasts, but the nuclei of osteoblasts showed no nuclear reactivity for *SOX6*.

## 3. Discussion

miR-132, which is located on chromosome 17p13.3, has been widely researched in recent years and is mainly implicated in neuropsychiatric disorders [25,26,33,34] and tumor progression in tumors, especially in osteosarcoma [29,30,31,32,35,36]. Recent evidence suggested that miRNA-132-3p inhibits osteoblast differentiation in simulated microgravity [27] and Type 2 Diabetes Mellitus-induced osteoporosis [28]. Furthermore, in a recent integrated microRNA-mRNA study, miR-132-3p was found to be one of the top 10 down-regulated miRNAs in ossified posterior longitudinal ligament (PLL) cells compared with normal PLL cells [37], thus suggesting that miR-132-3p may be involved in the process of ligament ossification. The present study provides the first evidence that miR-132-3p suppresses the osteogenic differentiation of LF cells. Our results showed that miR-132-3p was down-regulated during osteogenic differentiation. Further, inhibition of the expression of miR-132-3p promoted osteogenic differentiation, while miR-132-3p overexpression inhibited osteogenic differentiation. These findings suggest that miR-132-3p acts as a negative regulator of the osteogenic differentiation of LF cells.

It is well known that one miroRNA can regulate multiple target genes. Among the potential miR-132-3p targets predicted using Targetscan, three osteogenesis-related genes, *FOXO1*, *GDF5* and *SOX6*, were selected in this study. The dual luciferase reporter assays identified these three genes as direct targets of miR-132-3p. Furthermore, miR-132-3p overexpression resulted in their down-regulation at the protein level, whereas inhibition of miR-132-3p led to their up-regulation, suggesting that *FOXO1*, *GDF5* and *SOX6* are regulated by miR-132-3p during osteogenic differentiation. Though the regulatory connection between miR-132-3p and *FOXO1* has already been reported in gastric cancer cells [38], the direct targeting of miR-132-3p with *GDF5* and *SOX6* was newly discovered. Besides, miR-132-3p regulated osteoblast differentiation in osteoporosis by targeting EP300 [27] and SIRT1 [28]. Whether these two genes were involved in osteogenic differentiation of LF cells requires further study.

The pathological process of OLF involves the differentiation of fibroblasts into osteoblasts. Many cytokines, including transcription factors [39], growth factors [40] and inflammatory cytokines [41], have been reported to be involved in the ossification process. In the present study, *FOXO1*, *GDF5* and *SOX6* were all found to encourage the osteogenic differentiation of LF cells. We demonstrated that down-regulation of endogenous *FOXO1*, *GDF5* or *SOX6* suppressed osteogenesis to varying degrees, respectively. In addition, during the osteogenic differentiation of LF cells, the three osteogenesis-related genes were up-regulated, and the highest value of each gene expression was found at different times. The results indicated that these three genes may take effect in different stages of osteogenic differentiation and also suggested that miR-132-3p may regulate different genes in different stages of osteogenic differentiation. Location expressions of *FOXO1*, *GDF5* and *SOX6* were visually observed by immunohistochemistry. From the perspective of histopathology, the progression of OLF is viewed as a process of endochondral ossification. *FOXO1* and *GDF5* were positively expressed in both the cartilage and ossified area, while *SOX6* was only positively expressed in the cartilage area. The results were in agreement with the cell experiment.

*FOXO1* is the main member of the orkhead box O (FoxO) family expressed in bone and is a positive regulator of osteoblast differentiation [42,43,44]. *GDF5*, also called bone morphogenetic protein (BMP)-14 and cartilage-derived morphogenetic protein (CDMP)-1, is a member of the transforming growth factor β (TGF-β) superfamily and plays critical roles in organ development processes including bone, cartilage, ligament, and joint formation [45]. *SOX6*, a member of the D subfamily of sex determining region y-box (SRY-box)-related transcription factors, is a crucial transcription factor that regulates chondrogenesis and endochondral ossification [46]. A previous study showed that rhGDF-5 induces the osteogenic differentiation of LF cells through the activation of ERK1/2 and p38 mitogen-activated protein kinase (MAPK) [40]. However, the molecular mechanisms of *FOXO1* and *SOX6* in the osteogenic differentiation of LF cells were unclear. More research is required to understand the detailed mechanism of these osteogenesis-related genes in LF cells.

## 4. Materials and Methods

### 4.1. Patient Specimens

All experimental protocol involving humans (including the acquisition, processing and detection of the specimens) were approved by the Medical Scientific Research Ethics Committee of Peking University Third Hospital (PUTH-REC-SOP-06-3.0-A27, 2014003). All the methods were performed in accordance with relevant guidelines and regulations. TOLF patients who visited the orthopedic clinic and provided written informed consent for the study were utilized. Specialists diagnosed TOLF based on clinical symptoms and radiological examination as previously described [47]. Ligamentum flavum samples were obtained from TOLF patients during spinal surgery via en bloc resection of the lamina and ligamentum flavum as previously described [48].

### 4.2. Cell Cultures and Osteogenic Differentiation

Ligaments (approximately 0.5–1 cm^2^) were aseptically harvested from patients during surgery and rinsed with phosphate-buffered saline (PBS), while surrounding tissues were removed under a dissecting microscope to avoid possible osteogenic cell contamination. The collected ligaments were minced into approximately 0.5 mm^3^ pieces and digested using 0.25% trypsin, followed by 250 U/mL type I collagenase (Sigma-Aldrich, St. Louis, MO, USA). The specimen were washed with serum-containing medium and placed in 100-mm culturing dishes containing Dulbecco’s Modified Eagle′s medium (DMEM; GIBCO, Grand Island, NY, USA) supplemented with 10% fetal bovine serum (GIBCO), 100 U/mL penicillin G sodium and 100 mg/mL streptomycin sulfate in a humidified atmosphere with 5% CO_2_ at 37 °C. Explant-derived cells derived were harvested using 0.25% trypsin for further passaging, with passages (P) 2 and 3 used for subsequent experimentation. To induce osteogenic differentiation, cells were cultured in osteogenic medium consisting of DMEM supplemented with 50 µM ascorbic acid (Sigma-Aldrich), 10 mM β-glycerophosphate (Sigma-Aldrich) and 10−8 M dexamethasone (Sigma-Aldrich).

### 4.3. Quantitative Real-Time Polymerase Chain Reaction (qRT-PCR) Analysis

Total RNA was isolated using Trizol (Invitrogen, Carlsbad, CA, USA). Reverse transcription and qPCR for miR-132-3p were performed using a miDETECTA Track™ miRNA qRT-PCR Starter kit (RiboBio, Guangzhou, China) according to the manufacturer’s instructions on a BioRad IQ5 system. Each value was normalized to that of RnU6. Reverse transcription and qPCR for the mRNA levels of *FOXO1*, *GDF5* and *SOX6* were carried out as described previously [49]. Expression levels were normalized to glyceraldehyde 3-phosphate dehydrogenase (GAPDH) and relative gene expression levels were calculated using the 2-ΔΔ*C*_t_ method. All experiments were performed in triplicate. The primers were described in Table 1.

### 4.4. Western Blot Analysis

Cell lysates were obtained using RIPA lysis buffer (Beyotime, Shanghai, China) containing 10 mM phenylmethylsulphonylfluoride as a protease inhibitor (PMSF; Beyotime) and 50 µg of total protein was separated in a Bis-Tris polyacrylamide gel and transferred onto a nitrocellulose membrane. The membrane was then incubated in 5% bovine serum albumin (BSA) containing primary rabbit-anti-human polyclonal antibodies at 4 °C overnight. After incubating with horseradish peroxidase (HRP)-conjugated goat-anti-rabbit antibody at room for 1 h, protein was detected using electrochemiluminescence (ECL; Millipore, Darmstadt, Germany). The following primary rabbit-anti-human antibodies were used: anti-*FOXO1* (1:1000; ab39670, Abcam, Cambridge, MA, USA); anti-*GDF5* (1:1000; ab93855, Abcam); anti-*SOX6* (1:1000; ab30455, Abcam); anti-Runx2 (1:1000; ab23981, Abcam); anti-Sp7/Osterix (1:2000; ab22552, Abcam); anti-ALP (1:2000; ab95462, Abcam); anti-OCN (1:500; ab93876, Abcam); anti-OPN (1:1000; ab8448, Abcam); and anti-GAPDH (1:2500; ab9485, Abcam). The results of Western blots were quantified using image-J software (http://imagej.net) analysis.

### 4.5. Alkaline Phosphatase (ALP) Activity Assay and Alizarin Red Staining

Cells were seeded in six-well plates at a density of 1 × 10^5^ cells/well and cultured in osteogenic medium for 14 days. ALP activity was determined using an ALP activity staining kit (DE0004; Leagene Biotech, Beijing, China) and mineralization was assessed using an Alizarin Red S kit (DS0002; Leagene Biotech).

### 4.6. miRNA/siRNA Transfection

Synthetic miRNA and siRNA were purchased from RiboBio. ligamentum flavum cells were transfected with miRNA/siRNA using Lipofectamine^®^ 2000 Transfection Reagent (Life Technologies, New York, NY, USA), according to the manufacturer′s instructions.

MiR-132-3p mimics or miR-132-3p inhibitor (anti-miR-132-3p) were transfected into LF cells at a concentration of 20 nM with nonspecific microRNA (miR-NC) or nonspecific microRNA inhibitor (anti-miR-NC) used as negative controls. SiRNA targeting *FOXO1*, *GDF5* or *SOX6* were transfected at a concentration of 50 nM with non-targeting siRNA (siNC) used as negative control.

### 4.7. Luciferase Constructs and Reporter Assay

The DNA sequences of *FOXO1*, *GDF5* or *SOX6* 3′-UTR were amplified by PCR using HEK293T genomic DNA as a template. The amplified DNA sequences were inserted into pmiR-RB-REPORT™ vectors (RiboBio) to generate wild type (WT) *FOXO1*, *GDF5* or *SOX6* 3′-UTR, with a mutated (mut) *FOXO1*, *GDF5* or *SOX6* 3′-UTR luciferase vector generated using site-directed mutagenesis. For the reporter assay, HEK293T cells were cultured in a 96-well plate with 1.5 × 104 cells/well in 100 μL of culture medium/well for 24 h. Cells were then co-transfected with 50 nM miR-132-3p mimic or miR-NC and 100 ng of vector per well and cultured in fresh medium for an additional 48 h. The luciferase reporter assay was carried out using the Dual-Glo® Luciferase Assay System (Promega, Madison, WI, USA) according to the manufacturer’s instructions and luminescence was quantified using a Veritas™ 9100-002 luminometer (Promega).

### 4.8. Hematoxylin-Eosin (HE) Staining, Tissue-Specific Staining and Immunohistochemical (IHC) Analysis

Serial 5-mm-thick sections were prepared from paraffin-embedded specimens for staining.

Hematoxylin-eosin staining was performed in an autostainer machine (ST5010 XL; Leica Microsystems, Mannheim, Germany) using standard procedures.

Elastic Fibers staining kit for ligament, Alcian Blue staining kit for cartilage and Fast Green staining kit for bone were purchased from Leagene Biotech (DC0066; DB0060; DZ0046).

Sections for immunohistochemical staining were carried out as described previously [49]. The primary rabbit anti-human antibodies were: anti-*FOXO1* (1:200; ab39670, Abcam); anti-*GDF5* (1:200; ab93855, Abcam); anti-*SOX6* (1:200; ab30455, Abcam).

## 5. Conclusions

Overall, our study provides a comprehensive profiling of miR-132-3p as a novel regulator during the osteogenic differentiation of LF cells. The findings presented herein show that miR-132-3p can suppress osteogenic differentiation of LF cells by targeting *FOXO1*, *GDF5* and *SOX6*. Further, we demonstrated that *FOXO1*, *GDF5* and *SOX6* are expressed in LF cells and ossified ligamentum flavum tissues, and these osteogenesis-related genes may take effect at different stages of the osteogenic differentiation of LF cells. Our findings reveal a new mechanism of the TOLF pathological process and suggest that miR-132-3p and its target genes could possibly be viable therapeutic targets for TOLF and other skeletal disorders.

## Figures and Tables

**Table 1:** Primer sequences for quantitative real-time polymerase chain reaction (qRT-PCR).

Gene	Primer (5′–3′)
*FOXO1*	Fw: AAGCTCCCAAGTGACTTGGATG Rv: CTGCTCACTAACCCTCAGCCTGA
*GDF5*	Fw: AAAAGGACAGCTTCCCGGAG Rv: GCCTCCCTTTCTGTCAGCAT
*SOX6*	Fw: TCAACATGTGGCCTCCCATC Rv: GATGACAGAACGCTGTCCCA
*GAPDH*	Fw: TCAAGGCTGAGAACGGGAAG Rv: TGGACTCCACGACGTACTCA
miR-132-3p	RT: GTCGTATCCAGTGCAGGGTCCGAGGTATTCGCACTGGATACGACCGACCATG Fw: GCGCGCGTAACAGTCTACAGC Rv: GTCGTATCCAGTGCAGGGTCC
U6	RT: AAAATATGGAACGCTTCACGAATTTG Fw: CTCGCTTCGGCAGCACATATACT Rv: CGCTTCACGAATTTGCGTGT
